# ARTEMIN synergizes with TWIST1 to promote metastasis and poor survival outcome in patients with ER negative mammary carcinoma

**DOI:** 10.1186/bcr3054

**Published:** 2011-11-07

**Authors:** Arindam Banerjee, Zheng-Sheng Wu, PengXu Qian, Jian Kang, Vijay Pandey, Dong-Xu Liu, Tao Zhu, Peter E Lobie

**Affiliations:** 1 Liggins Institute, University of Auckland, 2-6 Park Avenue, Auckland, 1023, New Zealand; 2 Hefei National Laboratory for Physical Sciences at Microscale and School of Life Sciences, University of Science and Technology of China, Hefei, Anhui, 230027, P.R. China; 3 Department of Pathology, Anhui Medical University, Hefei, Anhui, 230032, P.R China; 4 Department of Pathology, Shanghai Medical College, Fudan University, Shanghai, 200032, P.R China; 5 Cancer Science Institute of Singapore and Department of Pharmacology, National University of Singapore, Centre for Life Sciences, #03-06C, 28 Medical Drive, 117456, Singapore

## Abstract

**Introduction:**

ARTEMIN (ARTN) is an estrogen regulated growth factor, the expression of which promotes resistance to antiestrogen therapies and predicts poorer survival outcome of patients with estrogen receptor (ER) positive mammary carcinoma (ER+MC) treated with tamoxifen. ARTN is also expressed in ER negative mammary carcinoma (ER-MC). Herein, we determined the role of ARTN in ER-MC and defined the mechanism of action producing poor patient prognosis.

**Methods:**

We modulated the expression of ARTN in two ER- (mesenchymal/claudin-low) mammary carcinoma cell lines (BT549 and MDA-MB-231) by forced expression or small interfering RNA (siRNA) mediated depletion. The effects of modulation of ARTN expression were examined by various *in vitro *measures of oncogenicity, including the expression of TWIST1 messenger RNA (mRNA) and protein. *In vitro *results were correlated to xenograft studies in immunodeficient mice. Co-expression of ARTN and TWIST1 and their association to poor survival outcome were examined in a cohort of patients with ER-MC. Pathway analysis was performed by pharmacological inhibition of phosphorylation of AKT (pAKT-Ser 473) or modulation of TWIST1 expression.

**Results:**

ARTN expression resulted in ER-MC cells with enhanced mesenchymal characteristics, including increased invasion and a gene expression profile consistent with enhanced mesenchymal phenotype. ARTN stimulated ER-MC cell anchorage independent and 3D matrigel growth, endothelial cell adhesion and transmigration of ER-MC cells through an endothelial cell barrier. Forced expression of ARTN produced a larger, locally invasive tumour mass with tumour emboli that produced distant metastasis. ARTN regulated TWIST1 expression in ER-MC cells and ARTN expression was significantly correlated to TWIST1 expression in a panel of mammary carcinoma cell lines and in a cohort of patients with ER-MC. Low expression of both ARTN and TWIST1 predicted 100% relapse free and overall survival in patients with ER-MC, whereas high expression of both ARTN and TWIST1 was associated with a poor survival outcome. ARTN stimulated an increase in TWIST1 expression via increased AKT activity. siRNA mediated depletion of TWIST1 abrogated ARTN stimulated cellular behaviour associated with metastasis, and forced expression of TWIST1 abrogated the functional effects of ARTN depletion.

**Conclusions:**

ARTN and TWIST1 synergize to produce a worse outcome in ER-MC and combined inhibition of ARTN and phosphatidylinositol 3-kinase/protein kinase B (PI3K/AKT) may therefore provide a novel therapeutic strategy in this subtype of mammary carcinoma.

## Introduction

Progression of mammary carcinoma is a complex process that involves aberrant regulation of multiple signalling pathways [1]. Determination of estrogen receptor (ER) status of invasive carcinomas before therapeutic intervention has become standard practice in the management of mammary carcinoma. Anti-estrogen therapy has proven to be successful for the treatment of ER-positive mammary carcinoma (ER+MC), which consequently generally has a better prognosis [2]. Conversely, ER-negative mammary carcinomas (ER-MC) are more aggressive and consistently associated with poorer prognosis [2]. Furthermore, based on molecular profiles, at least four different subtypes of mammary carcinoma (luminal A, luminal B, human epidermal growth receptor (HER)2-enriched and basal-like) have been identified [3]. Another mammary carcinoma subtype that has recently been delineated, termed claudin-low, exhibits high expression of genes involved in epithelial-mesenchymal transition (EMT), such as *SNAI1 *and *TWIST1*, and cancer stem cell-like features [4,5]. Compared with the luminal, HER2-enriched and basal-like subtypes, claudin-low tumors exhibit lower expression of ER, progesterone receptor (PR), HER2 and GATA3 and higher expression of mesenchymal, cell migration and angiogenesis genes [4]. Clinicopathological analyses also revealed that claudin-low tumors have poorer prognosis and may not be managed effectively with existing chemotherapy regimens [4]. Identification of novel and targetable molecular pathways responsible for propagating the poor prognosis in ER-MC is therefore warranted.

ARTEMIN (ARTN) is a member of the glial-cell line-derived neurotrophic factor (GDNF) family of ligands [6]. ARTN has been reported to promote mammary [7,8], endometrial [9], lung [10] and pancreatic [11] carcinoma progression. *ARTN *is an estrogen-regulated gene and it has been demonstrated that ARTN reduces the efficacy of anti-estrogens in ER+MC [8]. Furthermore, ARTN expression is correlated with decreased survival of patients with ER+MC treated with tamoxifen. Importantly, depletion or inhibition of ARTN partially restores tamoxifen sensitivity in tamoxifen-resistant mammary carcinoma cells [8].

TWIST1 belongs to the family of basic helix-loop-helix transcription factors originally found to modulate the expression of various target genes through canonical E-box responsive elements [12,13]. Increased expression of TWIST1 is observed in various human cancers [5,14,15] including ER-negative and claudin-low mammary carcinoma [4]. Evidence also indicates that TWIST1 contributes to cancer cell dissemination by promoting EMT and increasing cellular invasiveness [16,17]. Furthermore, a recent report suggests that TWIST1 interacts with several components of the Mi2/nucleosome remodelling and deacetylase (Mi2/NuRD) complex including metastasis-associated protein family member 2 (MTA2). MTA2 is a corepressor of ERα and increased MTA2 expression leads to estrogen-independent growth of mammary carcinoma cells during mammary carcinoma progression and metastasis [18].

Progression from estrogen dependence to estrogen independence (anti-estrogen resistance) in mammary carcinoma involves the altered expression of one or more estrogen-regulated gene networks [19,20]. In addition to ARTN stimulation of ER-transcriptional activity and the expression of estrogen-responsive genes, we have previously demonstrated that ARTN also promotes estrogen-independent growth of ER+MC cells [8]. ARTN may therefore possess a functional role in ER-MC. Indeed, in a cohort of patients [21], not only was ARTN expression correlated with worse survival outcome in ER+MC patients [8], it also predicted worse survival outcome in ER-MC patients. Such observations prompted us to examine the role of ARTN in ER-MC.

We report herein that ARTN stimulates oncogenicity and metastasis of ER-MC cells via expression of TWIST1 and that functional interaction between ARTN and TWIST1 promote a worse survival outcome in ER-MC.

## Materials and methods

### Cell culture

Cell lines used in this study were obtained from the American Type Culture Collection and cultured as recommended. BT549-ARTN and BT549-VEC cells have been previously described [7]. MDA-MB-231 cells were stably transfected with pIRESneo3-ARTN or the empty pIRESneo3 vector plasmids [7] to generate MDA-MB-231-ARTN and the control MDA-MB-231-VEC cells, respectively. Similarly, MDA-MB-231 and BT549 cells were stably transfected with pSilencer-siARTN B and pSilencer-CK plasmids [8] to obtain MDA-MB-231-siARTN, BT549-siARTN and MDA-MB-231-siCONT, BT549-siCONT cells, respectively. BT549 and MDA-MB-231 cell pairs were transiently transfected with 20 nM stealth siRNA TWIST1 or universal negative control (Invitrogen, Carlsbad, CA, USA) using Lipofectamine 2000™ for 24 hours and functional assays performed as described below.

### Reagents

Tetramethylrhodamine isothiocyanate phalloidin was purchased from Sigma (St. Louis, MO, USA). AKT inhibitor IV was purchased from Calbiochem (San Diego, CA, USA).

### Quantitative PCR

Quantitative PCR (qPCR) was performed as described earlier [8,22,23]. In addition *SNAI2 *primers used for qPCR were; forward 5'-TGTTTGCAAGATCTGCGGCAAG-3' and reverse 5'-TGACCTGTCTGCAAATGCTC-3'. *SPARC *primers used for qPCR were forward 5'-AGCACCCCATTGACGGGTA-3' and reverse: 5'-GGTCACAGGTCTCGAAAAAGC-3'. For the *in vivo *xenograft, the following primers were used: *hHPRT *forward 5'-TTCCTTGGTCAGGCAGTATAATCC-3' and reverse 5'-AGTCTGGCTTATATCCAACACTTCG-3' *mgapdh *forward 5'-CTCACTCAAGATTGTCAGCAATG-3' and reverse 5'-CACATTGGGGGTAGGAACAC-3'.

### Immunoblotting and immunofluorescence

Western blot analysis was performed as described earlier (Liu and Lobie, 2007 [7]) using the following antibodies: goat anti-ARTN polyclonal antibody (R&D Systems, Minneapolis, MN, USA), mouse anti-β-ACTIN monoclonal antibody (Sigma, St Louis, MO, USA), rabbit anti-TWIST1 polyclonal antibody (Santa Cruz, CA, USA), rabbit anti-Akt polyclonal antibody and rabbit anti-p-Akt polyclonal antibody (Cell Signaling Technology, Beverly, MA, USA). Immunofluorescent (IF) imaging was performed as previously described [9] using goat anti-TWIST1 antibody. F-actin was visualised and IF localisation was performed as previously described [9] using goat anti-TWIST1 antibody (Santa Cruz, CA, USA).

### Cell function assays

Soft agar colony formation assay, 3D cell growth, cell migration and invasion assays were performed as previously described [7]. For monolayer cell proliferation, 3,000 cells were seeded into 100 μl media (2% FBS) and every 24 hours viability was quantified by alamarBlue (Invitrogen, Carlsbad, CA, USA) as described previously [8]. Colony scattering assays were performed as previously described [23]. Endothelial cell adhesion assay was performed as described previously [24], with minor modifications, as 5 μM CFMDA cell tracker green (Invitrogen, Carlsbad, CA, USA) was used to label cells. Adherent cells were observed and photographed under a fluorescence microscope (Olympus, Tokyo, Japan). Fluorescence was measured with excitation wavelength at 492 nm and emission wavelength at 517 nm. The trans-migration assay was performed as described previously [25].

### Metastatic seeding assay

All animal work was conducted in accordance with a protocol approved by the institutional animal care and ethics committee. Tumor growth and metastasis assays were performed as mentioned earlier [7,26,27]. For direct tail vein injection, Balb/c nude mice (Shanghai Slaccas Co., Shanghai, China) (four to six weeks old) were used. A 1.5 × 10^6 ^sample of viable BT549-VEC and BT549-ARTN cells (*n *= 7 for each group) were washed and harvested in PBS and subsequently injected into the lateral tail vein in a volume of 0.1 ml. A 3 × 10^6 ^sample of viable MDA-MB-231-VEC and MDA-MB-231-ARTN cells were injected subcutaneously into the mammary fat pad of immunodeficient nude mice (*n *= 6 for each group). After four weeks, mice were euthanized and lungs and livers were surgically resected for histology. Tissue samples were either fixed in 4% paraformaldehyde-PBS (pH = 7.4), embedded in paraffin and 6 μm-thick sections cut (for histological studies with hematoxylin and eosin) or were frozen at -80°C in RNALater (Ambion, TX, USA) for RNA extraction and qPCR analysis.

### Histopathological analysis

Tissue samples were collected from 94 ER-negative female mammary carcinoma patients attending the First Affiliated Hospital of Anhui Medical University (Hefei, P. R. China) presenting between 2001 and 2002. Institutional ethics committee approval for the project was obtained before commencement of the study and was in compliance with the Helsinki Declaration. Written informed consent was obtained from all patients. Of 94 patients, 33 were HER-2 positive and the remaining 61 were HER-2 negative as described in the additional file 1. All 94 patients underwent radical mastectomy. All patients had no previous diagnosis of carcinoma, no distant metastasis at the time of diagnosis, and no evidence of disease within one month after primary surgery. All patients received adjuvant chemotherapy (CMF regimen: cyclophosphamide, 600 mg/m^2 ^iv bolus, d1,8; methotrexate, 40 mg/m^2 ^iv bolus, d1,8; 5-fluorouracil, 600 mg/m^2 ^iv infusion, d1,8; every four weeks for six cycles). Patients who had undergone chemotherapy or radiation therapy before surgery were excluded from this study. Of 94 patients, 76 possessed full follow-up data ranging from 5 to 64 months, with a median time of 60.0 months and a mean time of 44.7 months.

Immunohistochemical (IHC) analysis of paraffin-embedded specimens was performed as described previously [7]. In brief, 3 μm thick tissue microarray (TMA) sections were deparaffinised in xylene, rehydrated in a graded series of ethanol solutions, and heated in a microwave oven in 0.01 M sodium citrate buffer (pH 6.0) for 10 minutes for antigen retrieval. Anti-ARTN antibody (rabbit) (Abcam, CAM, UK) was used at 1:100 dilution. The scoring procedure is outlined in additional file 1.

### Statistics

All numerical data are expressed as mean ± standard error of the mean from a representative experiment performed in triplicate, and statistical significance was assessed by Student's *t*-test (*P *< 0.05 was considered as significant) using Microsoft Excel XP (bar graphs) unless otherwise indicated (chi-squared test). Cox regression analysis was performed to determine the association of ARTN and TWIST1 expression to the risk of relapse and death.

## Results

### Forced expression of ARTN altered the morphology of ER-MC cells *in vitro*

**Figure 1:**
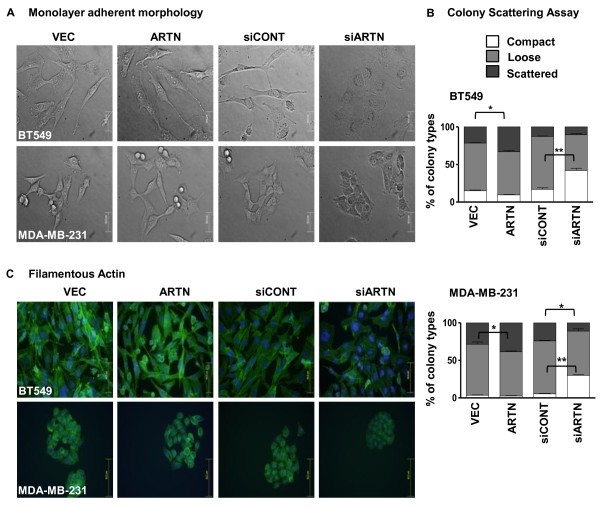
**ARTEMIN (ARTN) modulates cellular morphology of estrogen receptor (ER) negative mammary carcinoma (ER-MC) cells**. **(a) **Monolayer adherent morphology of BT549 and MDA-MB-231 cells with forced expression or depletion of ARTN. Cultures were imaged at approximately 40% confluence and under 400 × magnification (Bar represents 20 μm). **(b) **BT549-ARTN and MDA-MB-231-ARTN cells with their respective vector (VEC) cells and BT549-siARTN and MDA-MB-231-siARTN with their control (siCONT) cells were seeded at a very low cell density (1000 cell plated in 10 cm petri dish). Colonies formed by each cell types, were categorized and counted as per the extent of colony scattering. Percentages of each colony category in the total counts were plotted as indicated. **(c) **ARTN modulates organization of filamentous actin as visualised by fluorescein isothiocyanate (FITC) - phalloidin in BT549 and MDA-MB-231 cells with forced expression or depletion of ARTN compared with the respective control cells. Bar, 50 μm (f-actin staining for BT549-ARTN cells), 62.7 μm (f-actin staining for MDA-MB-231-ARTN cells). *, *P *< 0.05; **, *P *< 0.01.

To determine the functional consequences of ARTN expression in ER-MC, we generated stable cell clones with forced expression of ARTN in BT549 and in MDA-MB-231 cells (mesenchymal/claudin low cell lines) as previously described [7] [see additional file 2a]. Forced expression of ARTN in both BT549 and MDA-MB-231 cells resulted in a more scattered, elongated and mesenchymal cellular morphology in monolayer adherent culture as compared with the respective control VEC cells (Figure 1a) and as previously reported for BT549 cells [7]. In colony-scattering assays, we observed a significantly larger proportion of scattered cells (45% and 50% more than VEC cells) with forced expression of ARTN in both BT549 and MDA-MB-231 cells, respectively (Figure 1b) [see additional file 2b]. Visualisation of the filamentous actin (f-actin) cytoskeleton by fluorescence microscopy demonstrated that BT549-ARTN cells exhibited fewer, and more diffuse stress fibres with accumulation of f-actin at the cell periphery coinciding with the leading edges of the cell. In contrast, f-actin in BT549-VEC cells displayed a well-organised cortical ring near the cell periphery with a regular appearance of stress fibres similar to the parental cells. Similar reorganisation of the f-actin cytoskeleton was also observed in MDA-MB-231-ARTN cells (Figure 1c) [see additional file 2c]. When cultured in 3D matrigel, BT549-VEC cells produced circumscribed colonies, whereas BT549-ARTN cells exhibited more loose colonies as compared with the BT549-VEC cells [see additional file 2d]. Furthermore, a substantial number of BT549-ARTN cells spread from the main bulk of the colony, suggesting that ARTN promoted local invasive behavior. We also observed similar but less pronounced effects in MDA-MB-231-ARTN cells when cultured in 3D matrigel [see additional file 2d].

During the progression of carcinoma toward a less differentiated and more metastatic state, cells lose their epithelial characteristics and acquire a mesenchymal morphology, together with concomitant changes in gene expression [28]. Such a phenotypic conversion is referred to as EMT. Based on the changes in cell morphology observed with forced expression of ARTN, qPCR was therefore performed to determine the effects of forced expression of ARTN on the relative expression levels of key genes involved in EMT, cell invasion and metastasis. qPCR analysis of gene expression in BT549-ARTN and MDA-MB-231-ARTN cells demonstrated significantly decreased mRNA expression of the epithelial markers E-CADHERIN (*CDH1*), γ-CATENIN (*CTNNG*), and β-CATENIN (*CTNNB1)*. Decreased OCCLUDIN (*OCLN*) mRNA expression was also observed in BT549-ARTN cells but not in MDA-MB-231-ARTN cells. Increased mRNA expression of mesenchymal markers *TWIST1 *and *SPARC *was observed in BT549-ARTN and MDA-MB-231 cells, whereas VIMENTIN (*VIM*) and SLUG (*SNAI2*) mRNA expression was increased in BT549-ARTN cells (Table 1). The expression of *TWIST1 *mRNA in BT549-ARTN cells was increased 5.4-fold compared with control VEC cells and in MDA-MB-231-ARTN cells TWIST1 expression was increased by 4.5-fold compared with the VEC cells. Increased *TWIST1 *expression is correlated with an increased metastatic potential in mammary carcinoma cells [17,29]. Forced expression of ARTN consistently increased the mRNA level of the metastasis tumor-associated protein2 *(MTA2) *and *NFKB1 *in both cell pairs. Increased expression of *MTA2 *leads to estrogen independent growth of human mammary carcinoma cells [30] and increased expression of *NFKB1 *expression increases the invasive potential of carcinoma cells [31]. Furthermore, increased mRNA expression of *NME1*, *MMP9 *and *MYC *was observed in BT549-ARTN cells and *MMP2 *mRNA expression was increased in MDA-MB-231-ARTN cells, as compared with the respective control VEC cells. Thus, autocrine production of ARTN in ER-MC cells resulted in decreased expression of epithelial markers and increased expression of mesenchymal and metastatic related markers.

**Table 1:** Effects of forced expression of ARTEMIN (ARTN) in estrogen receptor (ER) negative mammary carcinoma (ER-MC) cells on expression of genes functionally involved in epithelial-mesenchymal transition (EMT) and metastatic progression

		BT549		MDA-MB-231	
Gene Function	Gene	Fold change	p-value	Fold change	p-value
**Epithelial**	*CTNNB1*	0.48	1.60E-03	0.47	1.25E-03
	*CTNNA1*	1.3	8.71E-03	0.68	5.80E-04
	*CTNNG*	0.52	8.23E-03	0.46	1.58E-02
	*OCLN*	0.35	9.71E-04	0.94	2.07E-02
	*CDH1*	0.12	4.93E-02	0.21	4.94E-03
**Mesenchymal**	*SNAI2*	1.68	6.26E-03	1.1	1.67E-02
	*VIM*	2.44	9.39E-04	1.23	6.79E-03
	*TWIST1*	5.49	3.98E-03	4.56	3.99E-03
	*SPARC*	1.82	2.03E-02	2.35	6.39E-03
**Metastatic**	*NME1*	2.7	1.57E-02	1.09	1.53E-03
	*PLAU*	1.02	4.40E-04	0.85	1.57E-03
	*MMP9*	2.11	3.64E-02	0.49	1.44E-03
	*MET*	0.85	1.70E-02	0.76	3.10E-03
	*MMP2*	1.04	2.07E-02	4.17	1.54E-03
	*MTA2*	3.02	8.75E-03	1.58	1.49E-02
	*MMP1*	1.29	4.36E-03	0.58	2.18E-02
	*NFKB1A*	2.23	7.19E-03	1.02	3.49E-03
	*NFKB1*	2.77	1.73E-02	1.7	3.96E-03
	*ERBB2*	1.24	1.71E-03	0.96	5.05E-03
	*MYC*	3.55	3.97E-04	0.95	1.40E-03

Results are represented as the fold change in messenger RNA (mRNA) levels in BT549-ARTN or MDA-MB-231-ARTN cells relative to the respective VEC cells. Values less than one indicates decreased expression and more than one indicates increased expression of the specific mRNA.

### Depletion of endogenous ARTN significantly alters the morphology of ER-MC cells *in vitro*

We next depleted BT549 and MDA-MB-231 cells of endogenous ARTN with small interfering RNA (siRNA) [8] [see additional file 2a] and examined the effects of depletion of ARTN on cellular morphology. In monolayer adherent culture, BT549-siARTN and MDA-MB-231-siARTN cells exhibited a coherent and relatively enhanced epithelial cellular morphology compared with cells with scrambled control siRNA (Figure 1a). In colony-scattering assays, we observed a significantly larger proportion of BT549-siARTN and MDA-MB-231-siARTN cells contained in compact colonies (200% and 480% more than siCONT cells) respectively (Figure 1b) [see additional file 2b]. Visualisation of f-actin by fluorescence microscopy demonstrated that cells with siRNA mediated depletion of ARTN possessed f-actin that was highly abundant and less diffuse within the cytoplasm of cells compared with the respective siCONT cells; pronounced cortical organisation of actin filaments and peripheral localisation of f-actin was rarely observed (Figure 1c) [see additional file 2c]. When cultured in 3D matrigel the morphology of colonies formed by BT549-siARTN cells in matrigel differed from that of BT549-siCONT cells. ARTN-depleted BT549-siARTN cells formed smaller and less numerous colonies, whereas the control BT549-siCONT cells formed colonies similar to BT549-VEC cells. We also observed similar effects in MDA-MB-231-siARTN cells, when cultured in 3D matrigel [see additional file 2d]. Therefore, siRNA mediated depletion of endogenous ARTN in ER-MC cells enhanced the epithelial characteristics of these cells.

### ARTN stimulates the oncogenicity and invasion of ER-MC cells *in vitro*

**Figure 2:**
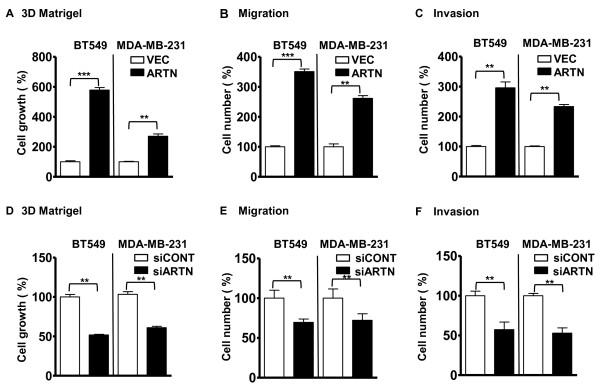
**ARTEMIN (ARTN) modulates cellular oncogenicity and motility of estrogen receptor (ER) negative mammary carcinoma (ER-MC) cells**. **(a) **Cell growth (number) in 3D matrigel. BT549-ARTN and MDA-MB-231-ARTN cells and their respective vector (VEC) cells were cultured in 3D matrigel for seven days. Cell growth was measured using alamarBlue. **(b) **Cell migration, and **(c) **Cell invasion for BT549-ARTN and MDA-MB-231-ARTN cells and their respective VEC cells were determined by use of transwell inserts as described in materials and methods. Respective VEC cells are presented as 100%. **(d) **Cell growth (number) in 3D matrigel. BT549-siARTN and MDA-MB-231-siARTN cells and their respective control (siCONT) cells were cultured in 3D matrigel for seven days. Cell growth was measured using alamarBlue. **(e) **Cell migration, and **(f) **Cell invasion for BT549-siARTN and MDA-MB-231-siARTN cells and their respective siCONT cells were determined by use of transwell inserts as described in materials and methods. Respective siCONT cells are presented as 100%. **, *P *< 0.01; ***, *P *< 0.001.

Increase in cellular invasiveness and resistance to anoikis are key components of metastasis [32]. Forced expression of ARTN significantly promoted monolayer proliferation, colony formation in soft agar [see additional files 3a and 3b], 3D matrigel cell growth, cell migration and invasion in transwell assays compared with their respective control BT549-VEC and MDA-MB-231-VEC cells (Figures 2a to 2c). BT549-ARTN and MDA-MB-231-ARTN cells showed a 35% and 22% increase in monolayer proliferation, 102% and 60% increase in soft agar colony formation, 479% and 170% increase in 3D matrigel growth, 249% and 160% increase in cell migration and 196% and 134% increase in cell invasion as compared with the control VEC cell lines, respectively and as previously reported in BT549 cells [7].

Concordantly, BT549-siARTN and MDA-MB-231-siARTN cells significantly inhibited monolayer proliferation by 23% and 17%, soft agar colony formation by 48% and 38% [see additional files 3c and 3d], 3D matrigel growth by 50% and 40%, cell migration by 31% and 27% and cell invasion by 38% and 40% as compared to the siCONT cells respectively (Figures 2d to 2f).

### ARTN enhances metastatic seeding of ER- MC cells *in vivo*

In our xenograft model, BT549 cells failed to form consistent tumor masses. Therefore, to determine whether ARTN contributes to the metastatic ability of ER-MC cells, we injected BT549-ARTN cells into the tail vein of immunodeficient nude mice and examined their ability to form pulmonary and hepatic metastasis compared with the control VEC cell line. Metastatic nodules were readily detectable by histology in the lungs of all mice (*n *= 7), four weeks after injection with BT549-ARTN cells, whereas pulmonary metastases were detected in only three of seven mice injected with BT549-VEC cells. The nodules formed in the lungs of animals injected with BT549-ARTN cells gave rise to, on average, more than three nodules per lung (Figureas 3a, left panel and 3b, left panel) as compared with less than one nodule per lung on average in animals with BT549-VEC cells. A higher frequency of liver metastasis was also observed in mice injected with BT549-ARTN cells (*n *= 4) compared with mice injected with BT549-VEC cells (*n *= 1) [see additional file 4a].

**Figure 3:**
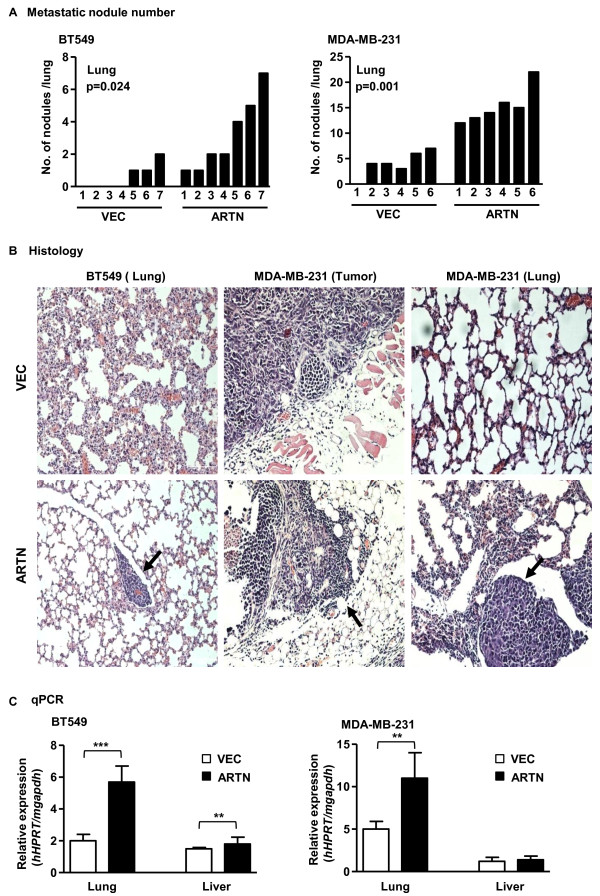
**ARTEMIN (ARTN) stimulates metastasis of estrogen receptor (ER) negative mammary carcinoma (ER-MC) cells**. **(a) **Number of mice with micrometastases per lung that received either lateral tail vein injection of BT549 (vector (VEC) or ARTN) cells (left panel) or from xenograft growth of MDA-MB-231(VEC or ARTN) cells (right panel) in the mammary fat pad. **(b) **H&E stained sections of lungs from mice that received lateral tail vein injection of BT549 (VEC or ARTN) cells (left panel), H&E staining of representative tumors from mice that received MDA-MB-231 (VEC or ARTN) cells in the mammary fat pad (middle panel) and H&E stained sections of lungs from the same group of mice (right panel). 200× magnification. Arrows indicate metastatic nodules. **(c) **Total RNA was isolated from lung and liver of individual mice that received either lateral tail vein injection of BT549 (VEC or ARTN) cells or mammary fat pad injection of MDA-MB-231 (VEC or ARTN) cells. Quantitative PCR (qPCR) was performed to measure the messenger RNA (mRNA) expression of human hypoxanthine-guanine phosphoribosyltransferase (*hHPRT*) in the lung and liver. Mouse glyceraldehyde 3-phosphate dehydrogenase (*mgapdh*) was used as an internal control. The relative expression of *hHPRT *vs *mgapdh *was calculated for each pair of BT549 and MDA-MB-231 cells. **, *P *< 0.01; ***, *P *< 0.001.

We extracted RNA from lung and liver of mice injected with BT549-ARTN cells or BT549-VEC cells and performed qPCR to quantitate the relative expression of human *HPRT (hHPRT) *mRNA in the respective organs. We observed that the level of *hHPRT *mRNA expression was increased three-fold in lung and 1.3-fold in liver in animals injected with BT549-ARTN cells compared with BT549-VEC cells. Mouse *gapdh *(*mgapdh*) was used as an internal control (Figure 3c, left panel).

Both MDA-MB-231-VEC and MDA-MB-231-ARTN cells formed palpable and measurable tumors when injected into the mammary fat pad of immunodeficient nude mice. Tumors formed by MDA-MB-231-ARTN cells were larger than those formed by control MDA-MB-231-VEC cells [see additional file 4b] (Figure 3b, middle panel). Pulmonary metastases were readily detectable by histology in the lungs of all mice (*n *= 6) four weeks after injection with MDA-MB-231-ARTN cells and in only five of the six mice injected with MDA-MB-231-VEC cells (Figure 3a, right panel and 3b, right panel). MDA-MB-231-ARTN cells gave rise to, on average, more than 15 nodules per section of lung as compared with approximately four nodules on average in animals with MDA-MB-231-VEC cells. We also extracted RNA from lung and liver of mice injected with MDA-MB-231-ARTN cells or MDA-MB-231-VEC cells and performed qPCR to quantitate the relative expression of *hHPRT *mRNA in the respective organs. We observed that the level of *hHPRT *mRNA expression was increased two-fold in lung and 1.2-fold in liver in animals injected with MDA-MB-231-ARTN cells compared with MDA-MB-231-VEC cells (Figure 3c, right panel). These results indicate that ARTN contributes to the metastatic potential of ER-MC cells.

### Expression and correlation of endogenous ARTN and TWIST1 in different mammary carcinoma cell lines

**Figure 4:**
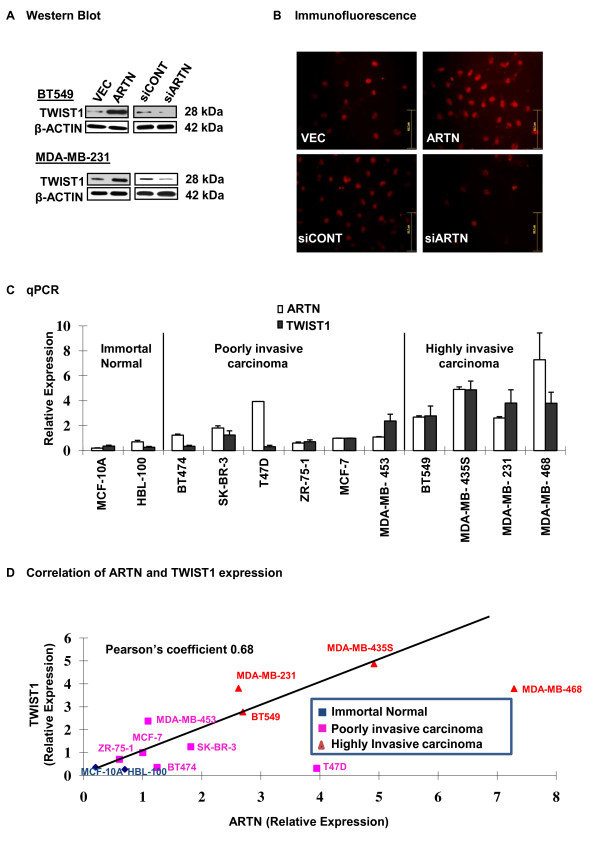
**ARTEMIN (ARTN) regulates expression of TWIST1 in estrogen receptor (ER) negative mammary carcinoma (ER-MC)**. **(a) **Western blot analyses for TWIST1 in BT549 and MDA-MB-231 cells with forced expression or depletion of ARTN. β-ACTIN was used as loading control for cell lysates. The sizes of detected protein bands in kiloDalton (kDa) are shown on the right. **(b) **TWIST1 expression in BT549-vector (VEC) and BT549-ARTN cells determined by immunofluorescent microscopy. Bar, 62.7 μm. **(c) **ARTN and TWIST1 messenger RNA (mRNA) levels were detected in two immortal but otherwise normal mammary epithelial cell lines and 10 different human mammary carcinoma cell lines with different invasive potential. Quantitative PCR (qPCR) was performed to measure the mRNA expression of TWIST1 and ARTN. Relative expression was calculated compared with the level of expression in MCF7 cells. **(d) **Pearson's correlation coefficient was determined between the mRNA expression of ARTN and TWIST1 in two immortal but otherwise normal mammary epithelial cell lines and 10 different human mammary carcinoma cell lines. TWIST1 expression was positively correlated with ARTN expression (rho = 0.68).

qPCR gene expression data demonstrated that forced expression of ARTN increased *TWIST1 *mRNA expression in both BT549 and MDA-MB-231 cells. Previous studies have demonstrated that TWIST1 is an important regulator of mammary carcinoma cell EMT and tumor metastasis [17]. To determine a potential molecular mechanism underlying ARTN stimulated invasion and metastasis of ER-MC cells, we therefore investigated the effects of forced expression and depletion of ARTN on the expression of TWIST1 protein. As observed in Figure 4a, TWIST1 protein expression was increased in both BT549-ARTN and MDA-MB-231-ARTN cells compared with respective control VEC cells. Concordantly, BT549-siARTN and MDA-MB-231-siARTN cells exhibited decreased expression of TWIST1 protein compared with the respective siCONT cells. IF microscopy of BT549-ARTN cells also demonstrated increased TWIST1 protein expression as compared with the BT549-VEC cells with TWIST1 being predominantly nuclear localized as previously reported [33]. Concordantly, BT549-siARTN cells exhibited decreased TWIST1 protein expression by IF as compared with the siCONT cells respectively (Figure 4b).

We next performed qPCR in two immortalised but otherwise normal human mammary epithelial cells and 10 different human mammary carcinoma cell lines of differing invasive potential. qPCR gene expression data showed low expression of both ARTN and TWIST1 mRNA in the two normal mammary epithelial cell lines. Both ARTN and TWIST1 mRNA were ubiquitously expressed across different mammary carcinoma cell lines and increased ARTN and TWIST1 mRNA expression correlated with a more invasive cell phenotype (Figure 4c). To further confirm the correlation of ARTN mRNA expression with TWIST1 mRNA expression, we compared the relative expression of ARTN mRNA and TWIST1 mRNA. We observed that TWIST1 mRNA expression was positively correlated with ARTN mRNA expression (Pearson coefficient: 0.68) (Figure 4d).

### Co-expression of ARTN and TWIST1 in ER-MC predicts survival outcome

**Figure 5:**
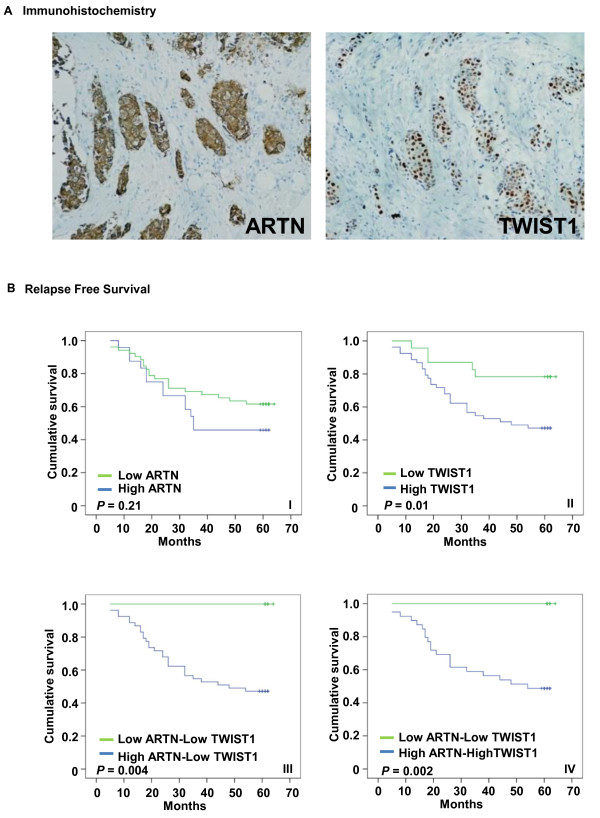
**Association of ARTEMIN (ARTN) and TWIST1 expression with survival outcome in estrogen receptor (ER) negative mammary carcinoma (ER-MC)**. **(a) **ARTN and TWIST1 expression detected by immunohistochemistry (IHC) in ER-MC samples. ARTN is predominantly localised to the cytoplasm whereas TWIST1 predominantly localized to the nucleus. 200× magnification. **(b) **Kaplan-Meier analysis of the association of ARTN expression (i), TWIST1 expression (ii), low-ARTN and low-TWIST1 vs high ARTN expression and low-TWIST1expression (iii) and low-ARTN and low-TWIST1 vs high ARTN and high TWIST1 expression (iv); relapse free survival (RFS) in ER-MC patients.

We next determined ARTN and TWIST1 protein expression in a cohort of human ER-MC by IHC and examined the association of expression with survival outcome (relapse free survival (RFS) and overall survival (OS)). IHC analysis showed that both ARTN and TWIST1 protein were highly expressed in ER-MC (Figure 5a). All 94 samples were positive for TWIST1 expression. We therefore stratified the samples according to the level of TWIST1 expression into two categories (low TWIST1 and high TWIST1 expression). ARTN immunoreactivity was stratified according to level of expression into four categories (negative (-), weak (+), moderate (++) and strong (+++) expression) [see additional file 1]. Of those tumors negative for ARTN expression, 60% exhibited strong TWIST1 expression. However, with increasing expression of ARTN in tumors, there was a concordant graded increase in TWIST1 expression with 86.7% of tumors with strong ARTN expression also exhibiting high TWIST1 expression. To further confirm the correlation of expression between TWIST1 and ARTN in mammary carcinoma, we compared the relationship by Spearman's rank correlation coefficient and found a significant positive value (Spearman correlation: r_s _= 0.21, *P *= 0.03) (Table 2). Hence, TWIST1 expression in ER-MC is partially correlated with ARTN expression.

**Table 2:** Correlation between ARTEMIN (ARTN) and TWIST1 expression in estrogen receptor (ER) negative mammary carcinoma (ER-MC).

	TWIST1 expression		
		Low	High
ARTN expression	Negative	14 (40%)	21 (60%)
	Weak	9 (31%)	20 (69%)
	Moderate	3 (20%)	12 (80%)
	Strong	2 (13.3%)	13 (86.7%)

**Spearman correlation: ***P *= 0.039 r_s _= 0.214. Expression parameters are described in details in the *Materials and methods *section.

We analyzed survival outcome of patients with tumors with either low or high ARTN or TWIST1. ARTN low tumors were defined as those with negative or weak expression of ARTN and ARTN high tumors exhibited moderate or strong expression of ARTN. Tumors with high ARTN expression tended to exhibit decreased RFS and OS of patients but this tendency did not reach significance in this cohort (Figure 5bi) [see additional file 5i]. A significant correlation of high TWIST1 expression to decreased RFS (*P *= 0.01) and OS (*P *= 0.03) of patients was observed (Figure 5bii) [see additional file 5i].

We subsequently analysed survival outcome in patients whose tumors exhibited both low ARTN and low TWIST1 expression compared with those that exhibited high ARTN and low TWIST1 expression. We observed that tumors with low TWIST1 and high ARTN were associated with decreased RFS (*P *= 0.004) and OS (*P *= 0.004) (Figure 5biii) [see additional file 5i] compared with those with low TWIST1 and low ARTN. Furthermore multivariate analysis demonstrated a significant association of ARTN high-TWIST1 low expression with decreased RFS (odds ratio: 3.4, 95% confidence interval (0.54-92.95): *P *= 0.03) and OS (odds ratio: 3.6, 95% confidence interval (0.55-99.84): *P *= 0.02) at five years compared with low ARTN-low TWIST1 expression (Table 3). Compared with tumors with low ARTN and low TWIST1 expression, tumors that exhibited both high ARTN and high TWIST1 expression were associated with worse RFS (*P *= 0.002) and OS (*P *= 0.005) (Figure 5biv) [see additional file 5i]. Furthermore, tumors with both low ARTN and low TWIST1 expression exhibited improved survival (RFS 100% (*P *= 0.002) and OS 100% (*P *= 0.005)), compared with low TWIST1 expression alone (RFS 78.3% (*P *= 0.01) and OS 78.3% (*P *= 0.03)) (Figure 5biv) [see additional file 5i] (Figure 5bii) [see additional file 5i]. In addition, multivariate analysis revealed a significant correlation of high ARTN-high TWIST1 expression to decreased RFS (odds ratio: 9.2, 95% confidence interval (0.51-163.76): *P *= 0.01) and OS (odds ratio: 9.2, 95% confidence interval (0.51-165.51): *P *= 0.01) at five years compared with low ARTN-low TWIST1 expression (Table 3).

**Table 3:** Multivariate analysis of the association of tumor ARTEMIN (ARTN) and TWIST1 expression with five-year relapse free (RFS) and overall survival (OS) in patients with estrogen receptor (ER) negative mammary carcinoma (ER-MC).

	RFS		OS	
	Odds ratio (95% CI)	*P*	Odds ratio (95% CI)	*P*
**ARTN-/ARTN+**	1.157 (0.971-1.461)	0.22	1.196 (0.943-1.518)	0.14
**TWIST1-/TWIST1+**	1.733 (1.076-2.791)	**0.024**	1.656 (1.026-2.674)	**0.039**
**Low ARTN-Low TWIST1/High ARTN-Low TWIST1**	3.420 (0.542-92.950)	**0.029**	3.676 (0.556-99.842)	**0.024**
**Low ARTN-Low TWIST1/High ARTN-High TWIST1**	9.202 (0.517-163.761)	**0.014**	9.268 (0.519-165.511)	**0.013**

CI, confidence interval.

### TWIST1 mediates ARTN-stimulated oncogenicity and invasion

**Figure 6:**
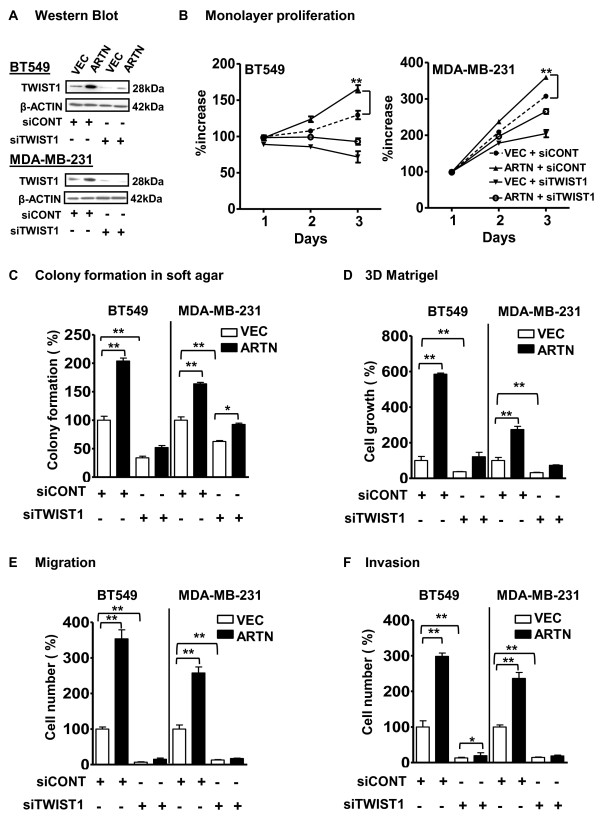
**ARTEMIN (ARTN) mediated oncogenicity is regulated by TWIST1 expression in estrogen receptor (ER) negative mammary carcinoma (ER-MC) cells**. **(a) **Western blot analysis for TWIST1 in BT549 and MDA-MB-231 cells with forced expression of ARTN ± TWIST1 small interfering RNA (siRNA) showing efficiency of siRNA-mediated depletion of TWIST1 protein. Universal negative control was used as an internal control. **(b) **Monolayer proliferation assay (2% fetal bovine serum media). **(c) **Colony formation in soft agar: Cells were cultured in 0.35% agar for 10 days. Cell growth was measured using alamarBlue. **(d) **Cell growth (number) in 3D matrigel: cells were cultured in 3D matrigel for seven days. Cell growth was detected using alamarBlue. **(e) **Cell migration and **(f) **Cell invasion were determined by use of transwell inserts as described in materials and methods. siRNA control-treated vector (VEC) cells presented as 100%. *, *P *< 0.05; **, *P *< 0.01.

To determine the functional significance of ARTN-stimulated TWIST1 expression we employed siRNA to selectively reduce TWIST1 expression in both BT549 and MDA-MB-231 cells with or without forced expression of ARTN. The siRNA targeting TWIST1 efficiently depleted TWIST1 protein expression in both cell pairs (Figure 6a) whereas transfection of scrambled siRNA (siCONT) had no effect on TWIST1 expression. Depletion of TWIST1 reduced the basal capacity for monolayer proliferation, colony formation in soft agar, 3D matrigel growth and migration and invasion in Transwell assays of BT549-VEC and MDA-MB-23-VEC cells as previously reported for mammary carcinoma cells [34]. Depletion of TWIST1 also prevented or largely abrogated the stimulatory effects of ARTN on monolayer proliferation, colony formation in soft agar, 3D matrigel growth, migration and invasion in transwell assays in BT549-ARTN and MDA-MB-231-ARTN cells (Figures 6b to 6f).

### ARTN stimulates mammary carcinoma cell attachment to, and migration through an endothelial cell layer

**Figure 7:**
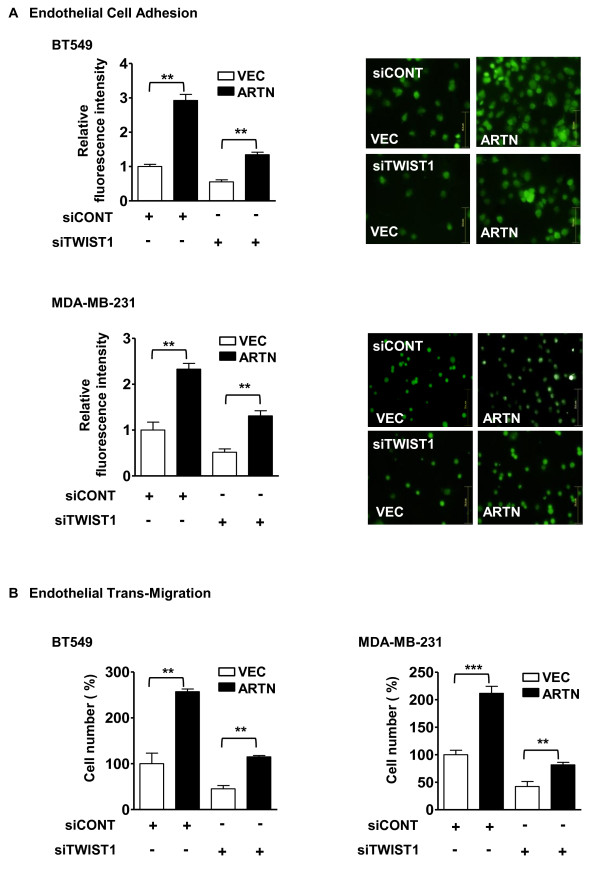
**TWIST1 mediates ARTEMIN (ARTN) stimulated attachment to, and migration through, an endothelial cell layer**. **(a) **Forced expression of ARTN in BT549 and MDA-MB-231 cells promoted adhesion to an endothelial cell layer: Human microvascular endothelial cells1 (HMEC1) were grown to 100% confluence in 96-well plates and 5-chloromethylfluorescein diacetate (CFMDA) cell tracker green (5 μM) labelled BT549-ARTN and MDA-MB-231-ARTN cells ± TWIST1 siRNA were seeded on top for one hour, washed and counted under a fluorescence microscope. Bar, 78.4 μm. **(b) **Forced expression of ARTN in BT549 and MDA-MB-231 cells promoted *in vitro *trans-migration activity through an endothelial cell layer: CFMDA labelled cells were seeded on top of HMEC1 cells grown 100% confluence on transwell inserts. After 14 hours invaded cells were counted under fluorescence microscope. siRNA control-treated vector (VEC) cells presented as 100%. **, *P *< 0.01; ***, *P *< 0.001.

As observed in Figure 7a, BT549-ARTN and MDA-MB-231-ARTN cells exhibited increased adherence to human microvascular endothelial cells (HMEC-1) by 2.8-fold and 2.3-fold as compared with their respective VEC control cells. Depletion of TWIST1 by siRNA significantly inhibited the ARTN stimulated enhancement of the adhesion of both cell pairs to the endothelial cell layer. BT549-ARTN and MDA-MB-231-ARTN cells also exhibited increased trans-migration through an endothelial cell layer by 2.5-fold and 2.0-fold as compared with their respective VEC control cells. TWIST1 depletion inhibited this trans-migration in both cell lines with forced expression of ARTN (Figure 7b).

### ARTN promoted oncogenicity is mediated via activation of AKT in ER-MC cells

**Figure 8:**
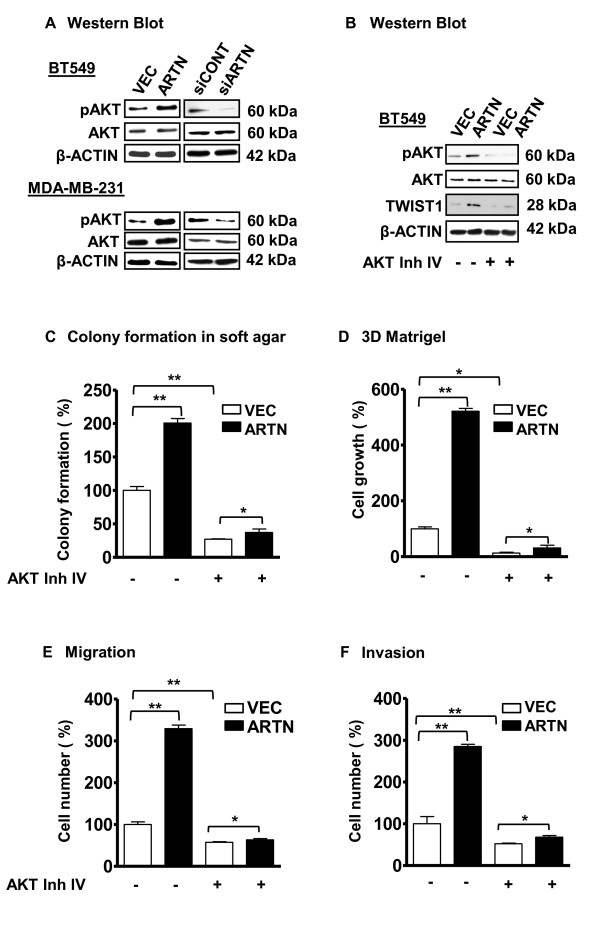
**ARTEMIN (ARTN) activates Protein kinase B (AKT) to increase TWIST1 expression in estrogen receptor (ER) negative mammary carcinoma (ER-MC)**. **(a) **Western blot analyses for phospho AKT (pAKT-Ser473) in BT549 and MDA-MB-231 cells with forced expression or depletion of ARTN. β-ACTIN was used as loading control for cell lysates. The sizes of detected protein bands in kiloDalton (kDa) are shown on the right. **(b) **Western blot was used to determine pAKT, AKT and TWIST1 protein levels in BT549-VEC and -ARTN cells ± AKT Inhibitor IV (10 μM). β-ACTIN was used as loading control. **(c) **Colony formation in soft agar. **(d) **Cell growth (number) in 3D matrigel. **(e) **Cell migration, and **(f) **Cell invasion was determined for BT549-VEC and -ARTN cells ± AKT inhibitor IV. Dimethyl sulfoxide (DMSO)-treated vector (VEC) cells are presented as 100%. *, *P *< 0.05; **, *P *< 0.01.

It has been previously reported that ARTN activates AKT to mediate its oncogenic effects in endometrial carcinoma cells [9]. The AKT signaling pathway has been reported to promote TWIST1 expression in different carcinoma cells [35,36]. Furthermore, Activation of AKT and TWIST1 in mammary carcinoma cells has previously been described [15,37]. We therefore determined total AKT protein and pAKT (Ser473) levels in BT549-ARTN and MDA-MB-231-ARTN cells compared with their control VEC cells. BT549-ARTN and MDA-MB-231-ARTN cells exhibited significantly increased phosphorylation of AKT (Ser473) compared with the respective VEC cells without alteration of the total AKT level. Concordantly, BT549-siARTN and MDA-MB-231-siARTN cells exhibited diminished AKT activation compared with the respective siCONT cells (Figure 8a).

We next evaluated whether signalling through AKT was required for ARTN stimulated oncogenicity and invasion in ER-MC cells. Treatment of BT549-VEC cells with AKT Inhibitor IV decreased the basal levels of pAKT but did not affect the total AKT level. ARTN stimulated activation of AKT was also decreased by AKT inhibitor IV without altering the expression level of total AKT (Figure 8b). AKT inhibitor IV decreased the basal level of TWIST1 expression in BT549-VEC cells and also significantly inhibited ARTN stimulated TWIST1 expression in BT549-ARTN cells. AKT Inhibitor IV reduced the basal capacity for colony formation in soft agar, 3D matrigel growth, migration and invasion in transwell assays of BT549-VEC and MDA-MB-23-VEC cells. AKT inhibitor IV also eliminated or largely abrogated the stimulatory effects of ARTN on colony formation in soft agar, 3D matrigel growth, migration and invasion in transwell assays in both BT549-ARTN and MDA-MB-231-ARTN cells (Figures 8c to 8f). Thus, ARTN utilizes AKT to increase TWIST1 expression and promote oncogenicity and invasiveness of ER-MC cells.

### ARTN activation of AKT is upstream of TWIST1 mediated oncogenicity in ER-MC cells

**Figure 9:**
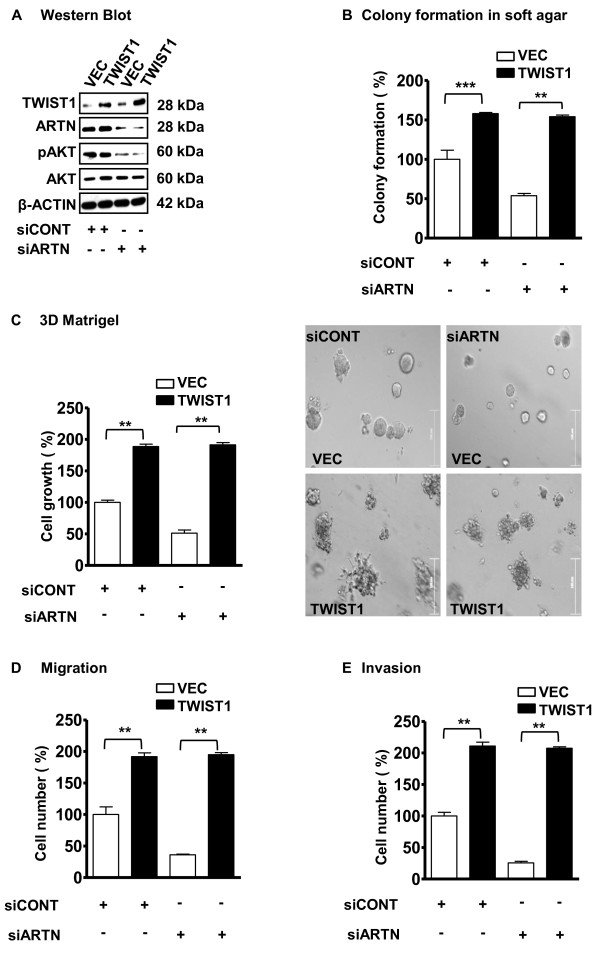
**TWIST1 is downstream of ARTEMIN (ARTN) stimulated Protein kinase B (AKT) activation in estrogen receptor (ER) negative mammary carcinoma (ER-MC)**. **(a) **Western blot analyses for ARTN, TWIST1, phospho AKT (pAKT-Ser473) and AKT in BT549 cells with forced expression of TWIST1 ± siARTN. β-ACTIN was used as loading control for cell lysates. The sizes of detected protein bands in kiloDalton (kDa) are shown on the right. **(b) **Colony formation in soft agar. **(c) **Cell growth (number) in 3D matrigel culture. **(d) **Cell migration, and **(e) **Cell invasion was determined for BT549-TWIST1 cells ± siARTN. siRNA control-treated vector (VEC) cells are presented as 100%. **, *P *< 0.01; ***, *P *< 0.001.

We determined if increased TWIST1 expression could substitute for the functional effects of ARTN in ER-MC cells. We generated stable cell clones with forced expression of TWIST1 in BT549 cells (Figure 9a) using a Myc tagged-TWIST1 expression vector [34]. Western blot analysis demonstrated that forced expression of TWIST1 in BT549 cells did not alter AKT expression or ARTN expression as compared with the VEC control cells. Depletion of ARTN by siRNA in BT549-VEC cells decreased the basal level of pAKT but did not alter total AKT or TWIST1 expression as compared with BT549-VEC cells with siCONT (Figure 9a). Depletion of ARTN by siRNA also decreased pAKT expression in BT549-TWIST1 cells, without altering AKT or TWIST1 expression as compared with siCONT cells. Thus, the activation of AKT precedes ARTN stimulated increases in TWIST1 expression.

We next analyzed the functional effects of siRNA mediated depletion of ARTN in BT549-TWIST1 cells. Forced expression of TWIST1 significantly promoted colony formation in soft agar, 3D matrigel growth, migration and invasion in transwell assays compared with the control BT549-VEC cells. BT549-TWIST1 cells exhibited a 60% increase in soft agar colony formation, 90% increase in 3D matrigel growth, 95% increase in cell migration and 110% increase in cell invasion (Figures 9b to 9e) as compared with the BT549-VEC cell line. Depletion of ARTN by siRNA decreased the basal level of colony formation in soft agar, 3D matrigel cell growth, migration and invasion in transwell assays in BT549-VEC control cells. However, in cells with forced expression of TWIST1, siRNA mediated depletion of ARTN exerted no effect. Thus, the functional effects of ARTN in ER-MC cells are mediated by increased TWIST1 expression.

## Discussion

Mammary carcinoma is a heterogeneous disease, which comprises a significant fraction of ER negative subclasses [38]. Given the poor disease prognosis and survival outcomes associated with ER-MC, delineation of prognostic markers to allow better identification and treatment are essential. Herein, we have demonstrated that ER-MC cells with mesenchymal/low-claudin subtype, express high levels of ARTN, which modulate the metastatic potential of cells by promotion of migratory and invasive behavior. Although ARTN is an estrogen-regulated gene [8], several reports have previously described the mechanisms of action of different growth factors to support estrogen independence during carcinoma tumor progression [39-42]. This is concordant with our previous report which demonstrated that ARTN promoted estrogen independent growth of ER+MC cells [8].

ARTN expression has previously been correlated with poorer survival outcome in mammary carcinoma overall [7] and in ER+MC patients treated with tamoxifen [8]. Herein, we have demonstrated that a lower expression of both ARTN and TWIST1 predicted a better survival outcome (100%) in ER-MC in contrast to either low expression of ARTN or TWIST1 alone, whereas high expression of both ARTN and TWIST1 predicted worse survival. That ARTN was only associated with survival outcome in ER-MC with low TWIST1 expression, and that forced expression of TWIST1 negated the effects of ARTN depletion, suggests that TWIST1 is the predominant mediator of ARTN function in ER-MC. Inhibition of ARTN would therefore not be effective as a single therapy in ER-MC with high TWIST1 expression. Acquired increased expression of TWIST1 would also negate the effects of inhibition of ARTN and presumably serve as a mechanism of resistance [43] to prolonged use of ARTN inhibitory strategies. However, based on the results presented herein, inhibition of ARTN, together with strategies to inhibit TWIST1 expression, such as inhibition of PI3K/AKT [44,45] would produce a more desirable outcome than use of either approach alone. Combined inhibition of ARTN and PI3K/AKT/TWIST1 could therefore represent a valuable therapeutic approach in ER-MC mammary carcinoma.

The prometastatic potential of TWIST1 in mammary carcinoma cells is well established [17,29,33]. Previously published reports delineating roles of transforming growth factor (TGF)-β and TWIST1 promoting EMT and metastasis [5] suggested that ARTN, a TFG-β superfamily member could also potentially regulate TWIST1 to promote EMT and metastasis [17]. TWIST1 can be regulated by multiple extracellular factors through PI3K/AKT, Wnt1 and NF-κB pathways in invasive carcinoma [46-48] concordant with our observations that activation of AKT by ARTN leads to increased expression of TWIST1 in ER-MC cells. Elevated expression of TWIST1 has been correlated with resistance to the microtubule targeting anticancer drugs taxol and vincristine [43] and TWIST1 expression is also increased in MC stem cell-like populations [49]. That increased ARTN expression induces genes associated with EMT and stimulates metastasis of ER-MC suggests that it may regulate a cancer stem cell-like sub-population of cells that possess an enhanced ability to metastasize to distant organs. Such a notion is concordant with a previous study [50] demonstrating that mammary carcinoma cell lines (including MDA-MB-231) contain functional cancer stem cells with metastatic capacity. In accordance, we have observed that forced expression of ARTN in ER-MC cells increased a sub-population of cells with cancer stem cell-like behavior and enhanced *in vivo *tumor initiating capability (manuscript in preparation). ARTN and TWIST1 may therefore functionally synergize in aspects of mammary carcinoma cell behavior other than EMT and metastasis reported herein.

## Conclusions

Although ARTN is an estrogen-regulated gene we have demonstrated that it also mediates oncogenic effects in ER-MC including cell invasion and metastasis. Functional involvement of GDNF family ligands has not been previously reported in ER-MC and the identified interactions with TWIST1 provide a novel therapeutic opportunity for this MC subtype. Furthermore, examination of the combined expression of ARTN and TWIST1 in ER-MC provides a powerful predictor of survival in these patients. It is therefore warranted that combinatorial therapeutic approaches to inhibit both ARTN signalling and PI3K/AKT, leading to reduced TWIST1, be considered to improve prognosis in patients with ER-MC.

## Abbreviations

ARTN: artemin; CFMDA: 5-chloromethylfluorescein diacetate; EMT: epithelial-mesenchymal transition; ER: estrogen receptor; ER+MC: estrogen positive mammary carcinoma; ER-MC: estrogen negative mammary carcinoma; F-actin: filamentous actin; FBS: fetal bovine serum; GDNF: glial-cell line-derived neurotrophic factor; HER2: human epidermal growth factor receptor; IF: immunofluorescence; IHC: immunohistochemistry; MTA: metastasis-associated protein family member; OS: overall survival; PBS: phosphate-buffered saline; PR: progesterone receptor; qPCR: quantitative real time PCR; RFS: relapse free survival; siRNA: small interfering RNA; TGF: transforming growth factor; TMA: tissue microarray.

## Competing interests

ZT and PEL received consultancies from Saratan Therapeutics Ltd (a biotech company formed around the potential to use Artemin as a target for breast cancer). Salaries of DXL and AB were part supported by Saratan Therapeutics Ltd. DXL and PEL are inventors on PCT/NZ2008/000152. PEL is an inventor on PCT/NZ2010/000207.

## Authors' contributions

PEL designed research, wrote the paper and analyzed the data. AB designed research, wrote the paper, performed research and analyzed the data. ZSW, PXQ performed research and analyzed data. JK and VP contributed to the execution of experiments. DXL, TZ analyzed data. All authors have read and approved the manuscript for publication.

## Supplementary Material

Additional file 1 **Histopathological Scoring **[51-53]. Click here for file

Additional file 2 **ARTN modulates the morphology of estrogen receptor-negative mammary carcinoma (ER-MC) cells**. Click here for file

Additional file 3 **ARTN modulates anchorage independent growth of estrogen receptor-negative mammary carcinoma (ER-MC) cells**. Click here for file

Additional file 4 **Xenograft growth of MDA-MB-231 cells with forced expression of ARTN in immunodeficient mice**. Click here for file

Additional file 5 **Association of ARTN and TWIST1 expression with overall survival outcome in estrogen receptor-negative mammary carcinoma (ER-MC) cells**. Click here for file
