# Supplementary material for: ARTEMIN synergizes with TWIST1 to promote metastasis and poor survival outcome in patients with ER negative mammary carcinoma
Source: Breast Cancer Res. 2011 Nov 7;13(6):R112. doi: 10.1186/bcr3054 (PMC3326554; doi:10.1186/bcr3054)
Supplement: Additional file 1 — Histopathological Scoring [51-53]. [file bcr3054-S1.DOC]

**additional file 1: *Histopathological Scoring***

The sections were scored as previously described [51, 52] on the basis of the staining intensity and the percentage of stained cells relative to the background. The staining intensity was scored as 0 ( 0% staining ), 1 ( <25% staining), 2 ( <75% staining ), and 3 ( >75% staining ) relative to the internal positive control; and the percentage of positive cells was scored as 0 (0%), 1 (1–25%), 2 (26–50%), 3 (51–75%), and 4 (>75%) of positive tumor cells. The staining score was calculated as the sum of staining intensity and percentage of positive tumor cells. For ARTN and HER-2 expression, tumors having final staining score of 0-1, 2-3, 4-5 and 6-7 considered as -, +, ++ and +++, respectively. TWIST1 antibody (SantaCruz, USA) was used at 1:200 dilution for immunohistochemistry. For TWIST1 expression, tumors with a final staining score of 6 or higher were considered to be high expression. ER expression was graded to negative (-) and positive (+), based on an assessment of the intensity of the reaction product and the percentage of positive cells: score –, no reactivity or nuclear reactivity in < 10% of tumor cells; +, nuclear reactivity is detected in > 10% of tumor cells as previously described [53].The scoring of sections was performed by two independent pathologists blinded towards clinicopathological data.
