# Supplementary material for: ARTEMIN synergizes with TWIST1 to promote metastasis and poor survival outcome in patients with ER negative mammary carcinoma
Source: Breast Cancer Res. 2011 Nov 7;13(6):R112. doi: 10.1186/bcr3054 (PMC3326554; doi:10.1186/bcr3054)
Supplement: Additional file 2 — ARTN modulates the morphology of estrogen receptor-negative mammary carcinoma (ER-MC) cells. [file bcr3054-S2.PPT]

## Slide 1
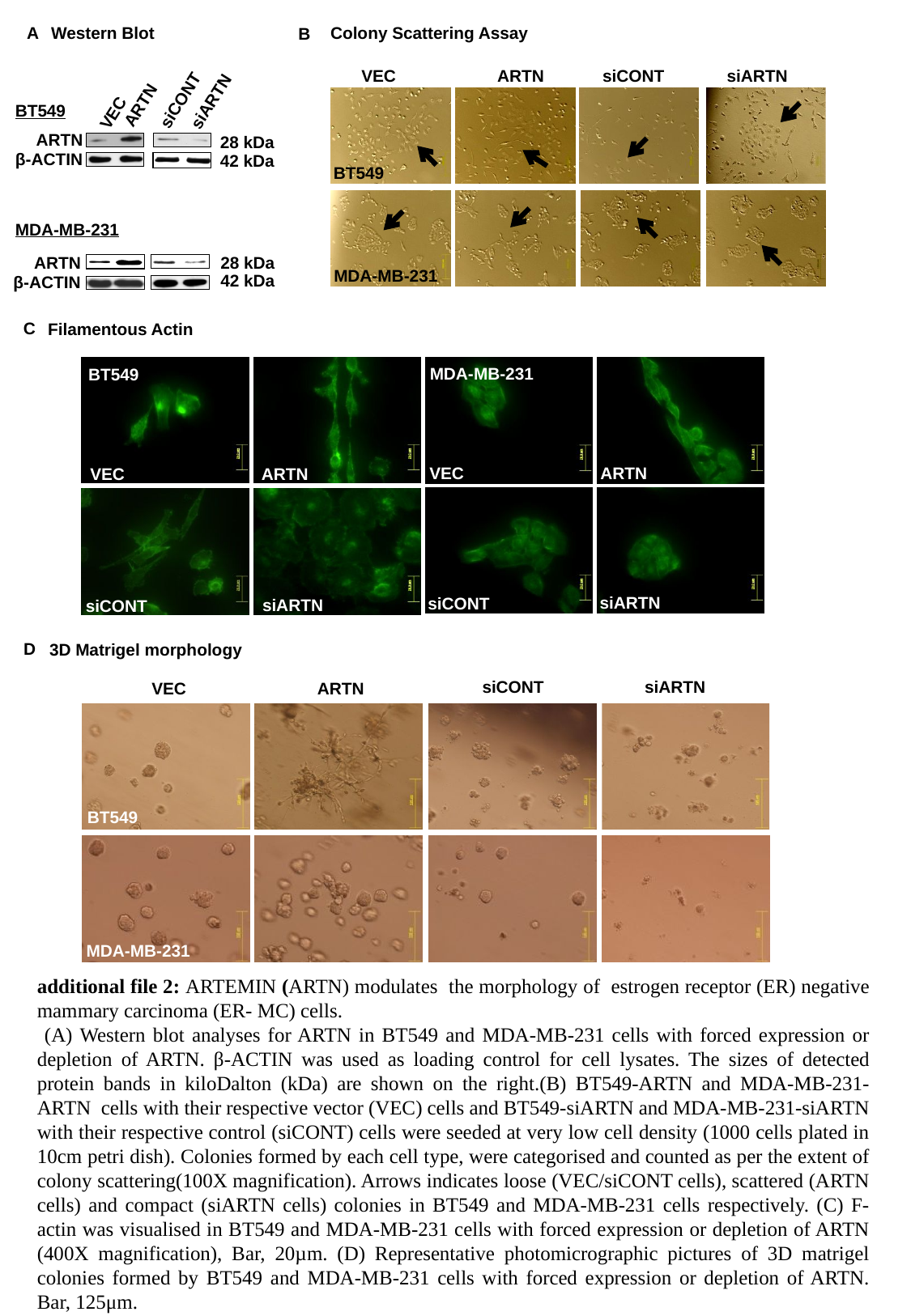

Colony Scattering Assay
A
Western Blot
B
siCONT
siARTN
ARTN
VEC
siCONT
siARTN
VEC
ARTN
BT549
ARTN
β-ACTIN
28 kDa
42 kDa
BT549
MDA-MB-231
28 kDa
ARTN
MDA-MB-231
42 kDa
β-ACTIN
C
Filamentous Actin
BT549
MDA-MB-231
VEC
ARTN
siARTN
siCONT
siARTN
siCONT
BT549
VEC
ARTN
D
3D Matrigel morphology
siCONT
siARTN
VEC
ARTN
BT549
MDA-MB-231
additional file 2: ARTEMIN (ARTN) modulates the morphology of estrogen receptor (ER) negative mammary carcinoma (ER- MC) cells.
 (A) Western blot analyses for ARTN in BT549 and MDA-MB-231 cells with forced expression or depletion of ARTN. β-ACTIN was used as loading control for cell lysates. The sizes of detected protein bands in kiloDalton (kDa) are shown on the right.(B) BT549-ARTN and MDA-MB-231-ARTN cells with their respective vector (VEC) cells and BT549-siARTN and MDA-MB-231-siARTN with their respective control (siCONT) cells were seeded at very low cell density (1000 cells plated in 10cm petri dish). Colonies formed by each cell type, were categorised and counted as per the extent of colony scattering(100X magnification). Arrows indicates loose (VEC/siCONT cells), scattered (ARTN cells) and compact (siARTN cells) colonies in BT549 and MDA-MB-231 cells respectively. (C) F-actin was visualised in BT549 and MDA-MB-231 cells with forced expression or depletion of ARTN (400X magnification), Bar, 20µm. (D) Representative photomicrographic pictures of 3D matrigel colonies formed by BT549 and MDA-MB-231 cells with forced expression or depletion of ARTN. Bar, 125μm.
