# Supplementary material for: ARTEMIN synergizes with TWIST1 to promote metastasis and poor survival outcome in patients with ER negative mammary carcinoma
Source: Breast Cancer Res. 2011 Nov 7;13(6):R112. doi: 10.1186/bcr3054 (PMC3326554; doi:10.1186/bcr3054)
Supplement: Additional file 3 — ARTN modulates anchorage independent growth of estrogen receptor-negative mammary carcinoma (ER-MC) cells. [file bcr3054-S3.PPT]

## Slide 1
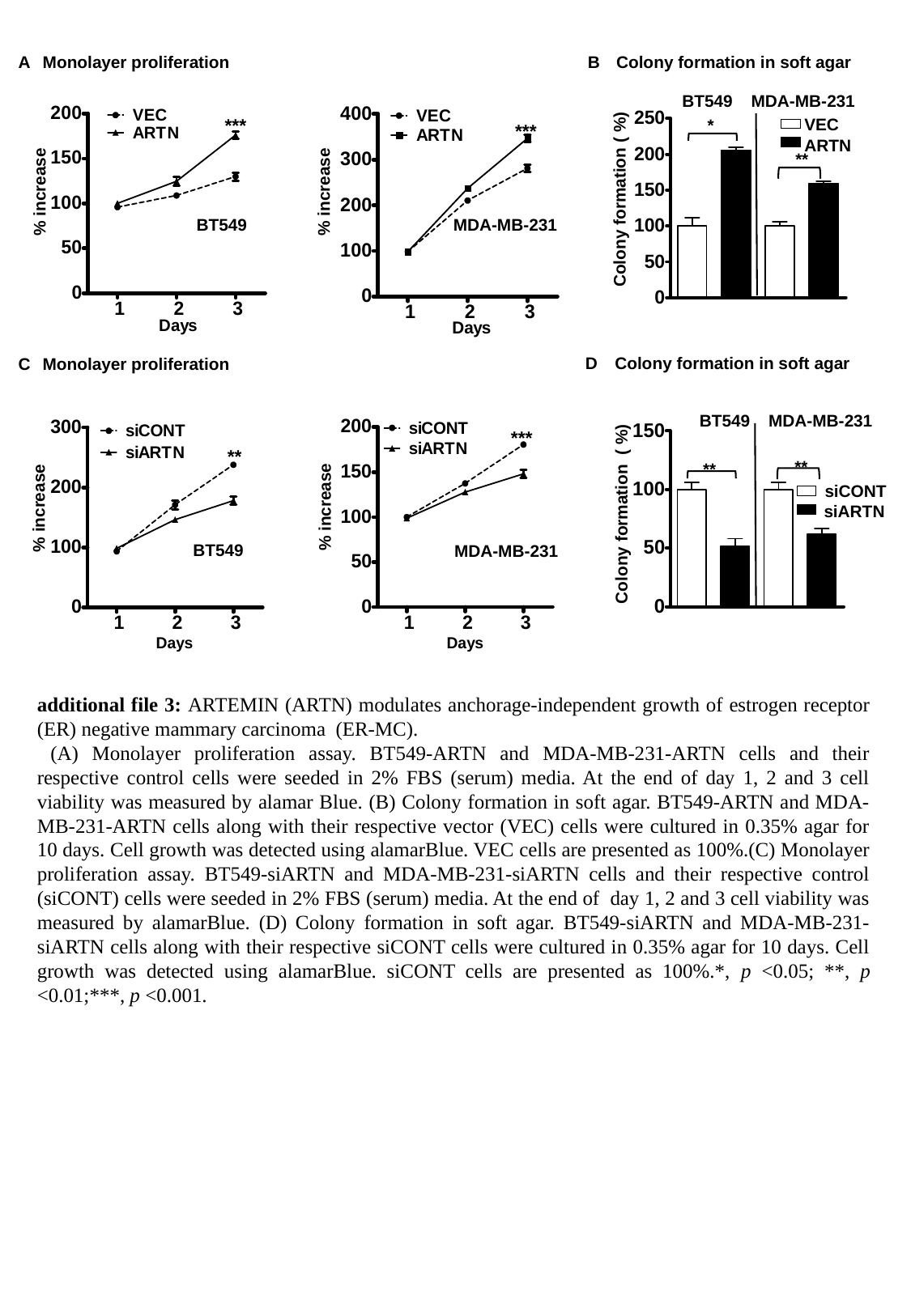

A
Monolayer proliferation
B
Colony formation in soft agar
 BT549 MDA-MB-231
*
**
***
VEC
ARTN
***
Colony formation ( %)
 % increase
 % increase
BT549
MDA-MB-231
D
Colony formation in soft agar
C
Monolayer proliferation
***
 % increase
MDA-MB-231
**
 % increase
BT549
 BT549 MDA-MB-231
**
**
 siCONT
siARTN
Colony formation ( %)
additional file 3: ARTEMIN (ARTN) modulates anchorage-independent growth of estrogen receptor (ER) negative mammary carcinoma (ER-MC).
 (A) Monolayer proliferation assay. BT549-ARTN and MDA-MB-231-ARTN cells and their respective control cells were seeded in 2% FBS (serum) media. At the end of day 1, 2 and 3 cell viability was measured by alamar Blue. (B) Colony formation in soft agar. BT549-ARTN and MDA-MB-231-ARTN cells along with their respective vector (VEC) cells were cultured in 0.35% agar for 10 days. Cell growth was detected using alamarBlue. VEC cells are presented as 100%.(C) Monolayer proliferation assay. BT549-siARTN and MDA-MB-231-siARTN cells and their respective control (siCONT) cells were seeded in 2% FBS (serum) media. At the end of day 1, 2 and 3 cell viability was measured by alamarBlue. (D) Colony formation in soft agar. BT549-siARTN and MDA-MB-231-siARTN cells along with their respective siCONT cells were cultured in 0.35% agar for 10 days. Cell growth was detected using alamarBlue. siCONT cells are presented as 100%.*, p <0.05; **, p <0.01;***, p <0.001.
