# Supplementary material for: ARTEMIN synergizes with TWIST1 to promote metastasis and poor survival outcome in patients with ER negative mammary carcinoma
Source: Breast Cancer Res. 2011 Nov 7;13(6):R112. doi: 10.1186/bcr3054 (PMC3326554; doi:10.1186/bcr3054)
Supplement: Additional file 4 — Xenograft growth of MDA-MB-231 cells with forced expression of ARTN in immunodeficient mice. [file bcr3054-S4.PPT]

## Slide 1
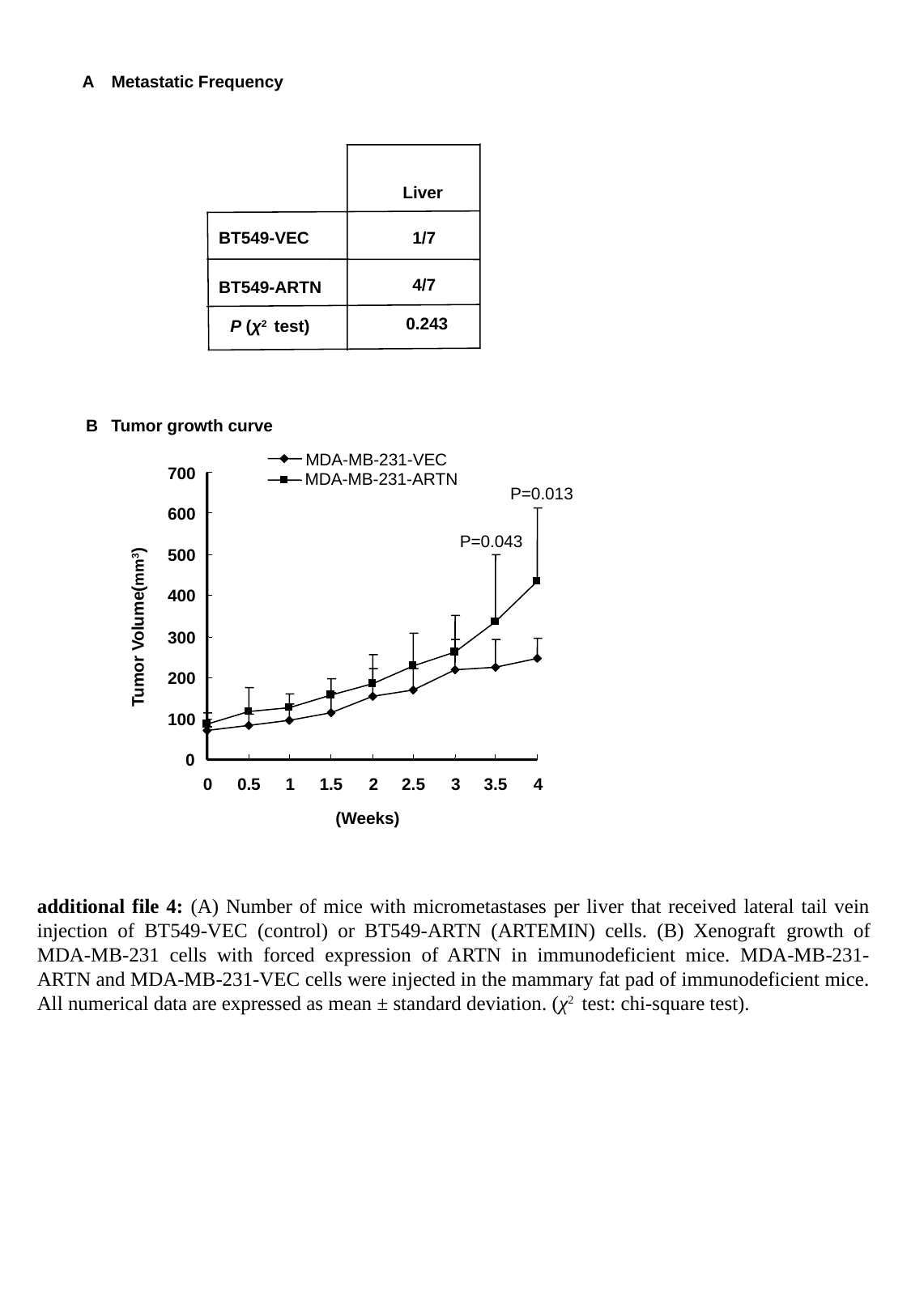

A
Metastatic Frequency
Liver
BT549-VEC
1/7
4/7
BT549-ARTN
0.243
P (χ2 test)
B
Tumor growth curve
MDA-MB-231-VEC
MDA-MB-231-ARTN
700
P=0.013
600
P=0.043
500
400
Tumor Volume(mm3)
300
200
100
0
0
0.5
1
1.5
2
2.5
3
3.5
4
(Weeks)
additional file 4: (A) Number of mice with micrometastases per liver that received lateral tail vein injection of BT549-VEC (control) or BT549-ARTN (ARTEMIN) cells. (B) Xenograft growth of MDA-MB-231 cells with forced expression of ARTN in immunodeficient mice. MDA-MB-231-ARTN and MDA-MB-231-VEC cells were injected in the mammary fat pad of immunodeficient mice. All numerical data are expressed as mean ± standard deviation. (χ2 test: chi-square test).
