# Supplementary material for: ARTEMIN synergizes with TWIST1 to promote metastasis and poor survival outcome in patients with ER negative mammary carcinoma
Source: Breast Cancer Res. 2011 Nov 7;13(6):R112. doi: 10.1186/bcr3054 (PMC3326554; doi:10.1186/bcr3054)
Supplement: Additional file 5 — Association of ARTN and TWIST1 expression with overall survival outcome in estrogen receptor-negative mammary carcinoma (ER-MC) cells. [file bcr3054-S5.PPT]

## Slide 1
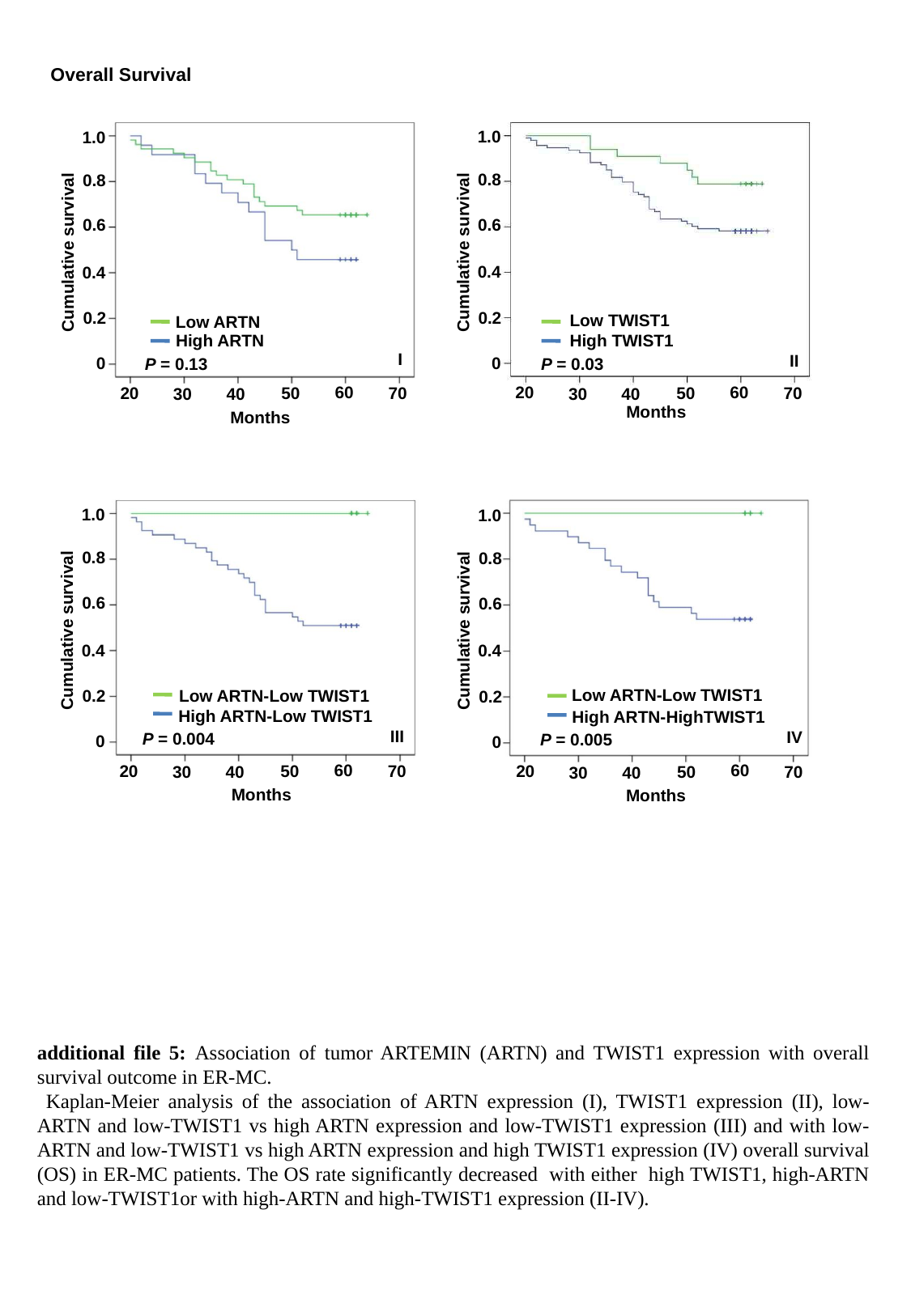

Cumulative survival
1.0
0.8
0.6
0.4
0.2
0
60
20
50
70
30
40
Cumulative survival
1.0
0.8
0.6
0.4
0.2
0
60
20
50
70
30
40
Overall Survival
Low TWIST1
Low ARTN
High ARTN
High TWIST1
I
II
P = 0.13
P = 0.03
Months
Months
Cumulative survival
1.0
0.8
0.6
0.4
0.2
0
60
20
50
70
30
40
Low ARTN-Low TWIST1
High ARTN-Low TWIST1
III
P = 0.004
Cumulative survival
1.0
0.8
0.6
0.4
0.2
0
60
20
50
70
30
40
Low ARTN-Low TWIST1
High ARTN-HighTWIST1
IV
P = 0.005
Months
Months
additional file 5: Association of tumor ARTEMIN (ARTN) and TWIST1 expression with overall survival outcome in ER-MC.
 Kaplan-Meier analysis of the association of ARTN expression (I), TWIST1 expression (II), low-ARTN and low-TWIST1 vs high ARTN expression and low-TWIST1 expression (III) and with low-ARTN and low-TWIST1 vs high ARTN expression and high TWIST1 expression (IV) overall survival (OS) in ER-MC patients. The OS rate significantly decreased with either high TWIST1, high-ARTN and low-TWIST1or with high-ARTN and high-TWIST1 expression (II-IV).
